# Bioactive Properties of *Tabebuia impetiginosa*-Based Phytopreparations and Phytoformulations: A Comparison between Extracts and Dietary Supplements

**DOI:** 10.3390/molecules201219885

**Published:** 2015-12-21

**Authors:** Tânia C. S. P. Pires, Maria Inês Dias, Ricardo C. Calhelha, Ana Maria Carvalho, Maria-João R. P. Queiroz, Lillian Barros, Isabel C. F. R. Ferreira

**Affiliations:** 1 Centro de Investigação de Montanha (CIMO), ESA, Instituto Politécnico de Bragança, Campus de Santa Apolónia, 1172, 5301-855 Bragança, Portugal; tania.pires@ipb.pt (T.C.S.P.P.); maria.ines@ipb.pt (M.I.D.); calhelha@ipb.pt (R.C.C.); anacarv@ipb.pt (A.M.C.); lillian@ipb.pt (L.B.); 2 Centro de Química, Universidade do Minho, Campus de Gualtar, 4710-057 Braga, Portugal; mjrpq@quimica.uminho.pt

**Keywords:** *Tabebuia impetiginosa*, methanolic extract/infusion/dietary supplements, antioxidants, cytotoxicity, chemical composition

## Abstract

*Tabebuia impetiginosa* (Mart. ex DC.) Standl. has been used in traditional medicine for many centuries, being nowadays marketed as dried plant material (inner bark) for infusions, pills, and syrups. The main objective of the present work was to validate its popular use through the bioactivity evaluation of the inner bark (methanolic extract and infusion) and of two different formulations (pills and syrup) also based on the same plant-material. The antioxidant activity was evaluated by in vitro assays testing free radical scavenging activity, reducing power and inhibition of lipid peroxidation in brain homogenates. The cytotoxicity was determined in four human tumor cell lines (MCF-7, NCI-H460, HeLa and HepG2, and also in non-tumor cells (porcine liver primary cells, PLP2)). Furthermore, the sample was chemically characterized regarding free sugars, organic acids, fatty acids, and tocopherols. Syrup and methanolic extract showed the highest antioxidant activity, related to their highest amount of phenolics and flavonoids. Methanolic extract was the only sample showing cytotoxic effects on the tested human tumor cell lines, but none of the samples showed toxicity in PLP2. Glucose and oxalic acid were, respectively, the most abundant sugar and organic acid in the sample. Unsaturated predominated over the saturated fatty acids, due to oleic, linoleic, and linolenic acids expression. α- and γ-Tocopherols were also identified and quantified. Overall, *T. impetiginosa* might be used in different phytoformulations, taking advantage of its interesting bioactive properties and chemical composition.

## 1. Introduction

*Tabebuia impetiginosa* (Mart. ex DC.) Standl., also known as *Handroanthus impetiginosus* (Mart. ex DC.) Mattos [1], (common and local names: pau d’arco, ipê-cavatã, ipê-comum, ipê-reto, ipê-rosa, ipê-roxo-damata, lapacho negro, pau d’arco-roxo, peúva or piúva) is a native species of the Bignoniaceae family from the Amazon rainforest and other tropical regions of South America and Latin America. The plant has been used in traditional medicine for many centuries, the inner bark being used in the treatment of pain, arthritis, inflammation of the prostate, fever, dysentery, boils, ulcers and to prevent different types of cancer [2,3]. Nowadays, it is marketed as dried plant material (bark) for infusions, pills, and syrups.

The chemical composition of this plant has been extensively studied and a variety of constituents have been isolated, such as furanonaphthoquinones, naphthoquinones, quinones, benzoic acids, cyclopentene dialdehydes, iridoids, and phenolic glycosides [4,5]. Its biological properties have been related mainly with the presence of naphthoquinones, which constitute the most prevalent active chemical group in the plant. Among the naphthoquinones, lapachol and β-lapachone are the two compounds that attracted the highest interest, being obtained from the bark [5]. Lapachol presents potent antiproliferative properties against various tumor cells [6], nonetheless, a phase I clinical trial was prematurely interrupted, due to the observance of secondary effects such as nausea and vomiting [7]. β-Lapachone proved to have a strong *in vitro* cytotoxic activity against several human and murine cell lines [8,9,10,11], but posterior negative results, obtained by *in vivo* studies with tumor-bearing mice [12], reduced the interest in further investigation with this compound.

Despite all the mentioned studies in individual compounds, the inner bark of *T. impetiginosa* continues to be used in several homemade preparations and in different dietary supplements, taking advantage of its chemical compounds, diversity, and potential synergisms. Therefore, the aim of this study was to validate the popular use of *T. impetiginosa* through the bioactivity evaluation of phytopreparations (methanolic extract and infusion) and phytoformulations (pills and syrup) based on its inner bark. This material was also chemically characterized in terms of individual hydrophilic and lipophilic compounds.

## 2. Results and Discussion

### 2.1. Chemical Characterization of T. impetigosa Inner Bark

The composition in hydrophilic and lipophilic compounds was determined and the results are shown in Table 1.

Oleic (C18:1n9), palmitic (C16:0), and linoleic (C18:2n6) acids were the most abundant fatty acids. The first one is nowadays considered as the preferred fatty acid for edible purposes, because it combines a hypocholesterolemic effect and a high oxidative stability [13]. Palmitic acid stimulates pro-inflammatory mechanisms through reactive oxygen species (ROS), while linoleic acid has been showing protective effects against cancer, obesity, diabetes, and atherosclerosis in animal studies and in different human cell lines [14]. Dietary fatty acids are increasingly recognized as major biologic regulators and have properties that relate to health outcomes and disease [14].

Fructose, glucose, and sucrose were the three free sugars identified in *T. impetigosa*, glucose being the most abundant and sucrose being the least abundant sugar. Oxalic, citric, and succinic acids were also identified and quantified, oxalic acid being the most abundant one (Table 1).

Oxalic acid is a common constituent of plants, and several species, including some crop plants. The most striking chemical impact of oxalic acid is its strong chelating ability with multivalent cations, being considered as an antinutrient due to its inhibitory effect on mineral bioavailability. However, this substance may function as a pH regulator and osmoregulator in plants [15]. Malic and fumaric acids were found only in trace amounts. The presence of free sugars and organic acids may be related with some bioactivities, namely antioxidants, as previously described by Roriz *et al.* [16] and Carocho *et al.* [17].

Regarding tocopherols, only α- and γ- tocopherol isoforms were detected. α-Tocopherol was, by far, the most abundant vitamer (Table 1). Considering its antioxidant potential and various functions at the molecular level, α-tocopherol can reduce the risk of cardiovascular diseases (eliminating reactive oxygen species, inhibiting lipid peroxidation, and attenuating inflammatory reactions) and neurodegenerative disorders, particularly in Alzheimer’s disease [18,19,20]. To the best of our knowledge this is the first report regarding the identification and quantification of these individual chemical compounds in *T. impetigosa.*

**Table 1:** Individual compounds in *T. impetigosa* inner bark (Mean ± SD).

Fatty Acids (Relative Percentage)		Free Sugars (g/100 g)	
C4:0	2.31 ± 0.28	Fructose	0.25 ± 0.01
C6:0	0.99 ± 0.01	Glucose	0.53 ± 0.04
C8:0	0.79 ± 0.01	Sucrose	0.10 ± 0.01
C10:0	1.76 ± 0.04	Total	0.88 ± 0.04
C12:0	1.23 ± 0.03	**Organic Acids (g/100 g)**	
C13:0	0.050 ± 0.001	Oxalic acid	0.21 ± 0.01
C14:0	3.35 ± 0.02	Malic acid	tr
C15:0	0.93 ± 0.01	Citric acid	0.11 ± 0.02
C16:0	17.86 ± 0.16	Succinic acid	0.15 ± 0.02
C16:1	0.12 ± 0.03	Fumaric acid	tr
C17:0	2.28 ± 0.13	Total	0.47 ± 0.05
C18:0	7.38 ± 0.13	**Tocopherols (mg/100 g)**	
C18:1n9	32.51 ± 0.32	α-Tocopherol	2.07 ± 0.17
C18:2n6	11.00 ± 0.02	γ-Tocopherol	0.24 ± 0.01
C18:3n3	9.40 ± 0.06	Total	2.31 ± 0.17
C20:0	2.34 ± 0.09		
C20:1	0.87 ± 0.09		
C20:3n3+C21:0	0.45 ± 0.04		
C22:0	1.52 ± 0.14		
C23:0	0.91 ± 0.01		
C24:0	1.97 ± 0.17		
SFA	47.68 ± 0.16		
MUFA	33.49 ± 0.01		
PUFA	18.83 ± 0.18		

The results are expressed in dry weight basis (dw); tr—races. SFA—saturated fatty acids; MUFA—monounsaturated fatty acids; PUFA—polyunsaturated fatty acids; Caproic acid (C6:0); Caprylic acid (C8:0); Capric acid (C10:0); Lauric acid (C12:0); Tridecanoic acid (C13:0); Myristic acid (C14:0); Pentadecanoic acid (C15:0); Palmitic acid (C16:0); Palmitoleic acid (C16:1); Heptadecanoic acid (C17:0); Stearic acid (C18:0); Oleic acid (C18:1n9); Linoleic acid (C18:2n6); α-Linolenic acid (C18:3n3); Arachidic acid (C20:0); Eicosenoic acid (C20:1); cis-11, 14, 17-Eicosatrienoic acid and Heneicosanoic acid (C20:3n3 + C21:0); Behenic acid (C22:0); Tricosylic acid (C23:0); Lignoceric acid (C24:0).

### 2.2. Antioxidant Properties of T. impetigosa Extracts and Dietary Supplements

The antioxidant properties of different dietary supplements based on *T. impetigosa* (syrup and pills) and of the different extracts prepared from the inner bark (infusion and methanolic extract) were compared (Table 2).

**Table 2:** Antioxidant properties of *T. impetigosa* extracts and dietary supplements (Mean ± SD).

**Antioxidant Activity (EC_50_, mg/mL)**	**Extract**	**Infusion**	**Pills**	**Syrup**
DPPH scavenging activity	0.68 ± 0.03 ^c^	16.68 ± 0.58 ^a^	5.63 ± 0.19 ^b^	0.30 ± 0.05 ^d^
Reducing power	0.27 ± 0.01 ^c^	6.78 ± 0.84 ^a^	3.45 ± 0.03 ^b^	0.26 ± 0.01 ^c^
β-Carotene bleaching inhibition	0.23 ± 0.04 ^c^	10.72 ± 3.53 ^a^	3.37 ± 0.83 ^b^	0.26 ± 0.02 ^c^
TBARS inhibition	0.14 ± 0.01 ^c^	1.87 ± 0.02 ^a^	1.60 ± 0.02 ^b^	0.02 ± 0.001 ^d^
**Antioxidant Compounds**	**Extract**	**Infusion**	**Pills **	**Syrup**
Phenolics (mg GAE/g extract)	247.50 ± 8.93 ^a^	8.11 ± 0.24 ^d^	14.54 ± 0.90 ^c^	29.43 ± 0.56 ^b^
Flavonoids (mg CE/g extract)	71.12 ± 4.42 ^a^	1.67 ± 0.02 ^d^	4.32 ± 0.13 ^c^	9.31 ± 0.12 ^b^

The antioxidant activity was expressed as EC_50_ values, what means that higher values correspond to lower reducing power or antioxidant potential. EC_50_: Sample concentration corresponding to 50% of antioxidant activity or 0.5 of absorbance in reducing power assay. Trolox EC_50_ values: 41 µg/mL (reducing power), 42 µg/mL (DPPH scavenging activity), 18 µg/mL (β-carotene bleaching inhibition) and 23 µg/mL (TBARS inhibition). In each row, different letters mean significant differences between species (*p <* 0.05).

The syrup and the methanolic extract revealed the highest antioxidant activity, while the infusion gave the lowest activity. It should be noted that the propyl gallate present in the syrup might have an antioxidant effect and therefore could have influence in the results. Our research group reported opposing results in a study with *Ginkgo biloba* L. in which infusions showed higher antioxidant activity than dietary supplements [21]. This result could be attributed to the very high amounts of phenolics found in Ginkgo dietary supplements (461.45–396.98 mg GAE/g) in comparison with the ones obtained in the present study for *T. impetigosa* syrup and pills (29.43–14.54 mg GAE/g). Another study [22] revealed a decrease in the bioactive compounds present in the infusion in comparison with the methanolic extracts, and these findings could be related to a degradation caused by the heat involved in infusion preparation procedure. According to these authors [22], phenolic compounds are unstable and easily became non-antioxidative under heating; thus, heat could destroy the structures of polyphenols and cause a decrease in their antioxidant activity.

The highest antioxidant activity exhibited by the methanolic extract and the syrup of *T. impetigosa* in all the assays could be related with the highest content of total phenolics observed in these samples, which is also supported by the literature for different plant species [23].

### 2.3. Cytotoxic Properties of T. impetigosa Extracts and Dietary Supplements

The methanolic extract showed inhibitory effect on the growth of different human tumor cell lines (MCF-7, NCI-H460, HeLa, and HepG2), being more effective against the non-small lung carcinoma cell line (NCI-H460) (Table 3).

**Table 3:** Cytotoxicity properties of *T. impetigosa* extracts and dietary supplements (Mean ± SD).

**Human Tumor Cell Lines (GI_50_ Values, µg/mL)**	**Extract**	**Infusion**	**Pills**	**Syrup**
MCF-7 (breast carcinoma)	110.76 ± 5.33	>400	>400	>400
NCI-H460 (lung carcinoma)	76.67 ± 7.09	>400	>400	>400
HeLa (cervical carcinoma)	93.18 ± 1.46	>400	>400	>400
HepG2 (hepatocellular carcinoma)	83.61 ± 6.61	>400	>400	>400
**Non-Tumor cells (GI_50_ Value, µg/mL)**	**Extract**	**Infusion**	**Pills**	**Syrup**
PLP2 (porcine liver primary cells)	>400	>400	>400	>400

GI_50_ values correspond to the sample concentration achieving 50% of growth inhibition in human tumor cell lines or in liver primary culture PLP2. Ellipticine GI_50_ values: 1.21 µg/mL (MCF-7), 1.03 µg/mL (NCI-H460), 0.91 µg/mL (HeLa), 1.10 µg/mL (HepG2) and 2.29 µg/mL (PLP2).

The other samples (infusion, pills and syrup) did not show any effect on the tested cell lines, up to 400 μg/mL. None of the samples showed toxicity to non-tumor cells (primary cultures of liver cells, PLP2). Junior *et al.* [24] correlated the antitumor activity of *T. Impetigosa* with the presence of quinones (lapachol and β-lapachone) in its constitution. Due to its structure, nephthoquinones could present biological activity leading to some synergistic effects between compounds explaining the cytotoxic effects of the extracts. Lapachol has also been demonstrated to reduce the number of tumors caused by doxorubicin in Drosophila melanogaster heterozygous for the tumor suppressor gene wts [25]. In addition to its antiproliferative activity in cancer cells, lapachol also decreases the invasion of HeLa cells and could therefore represent an interesting scaffold for the development of novel anti-metastatic compounds [26]. Lapachol induced anti-metastatic effects have also been demonstrated by Maeda *et al.* [27].

## 3. Materials and Methods

### 3.1. Samples

*T. impetiginosa* samples were purchased in an herbal shop in Bragança (Portugal), in three different forms: dried material of the inner bark for preparing infusions (botanical identification of the plant species was confirmed) and dietary supplements (pills and syrup). The pills were composed by the following ingredients: *T. impetiginosa* inner bark dry extract (5:1), microcrystalline cellulose (diluent), modified corn starch (binder), anhydrous dibasic calcium phosphate, dioxide silicon (anticaking agent), and magnesium stearate (lubricant). The syrup consisted of purified water, *T. impetiginosa* inner bark extract (10%), citric acid (acidity regulator), potassium sorbate, sodium benzoate and propyl gallate (preservative), aspartame, cyclamate sodium, and sodium saccharin (sweetener).

### 3.2. Standards and Reagents

Acetonitrile 99.9%, *n*-hexane 95%, and ethyl acetate 99.8% were of HPLC grade from Fisher Scientific (Lisbon, Portugal). The fatty acids methyl ester (FAME) standard mixture (standard 47885-U) was purchased from Sigma (St. Louis, MO, USA), as also were other individual fatty acid isomers, l-ascorbic acid, trolox (6-hydroxy-2,5,7,8-tetramethylchroman-2-carboxylic acid), ellipticine, phosphate buffered saline (PBS), acetic acid, sulforhodamine B (SRB), trichloroacetic acid (TCA), Tris, gallic acid, (+)-catechin, sugar and organic acid standards. Tocol (50 mg/mL) and tocopherol (α-, β-, γ-, and δ-isoforms) standards were purchased from Matreya (Pleasant Gap, PA, USA). 2,2-Diphenyl-1-picrylhydrazyl (DPPH) was obtained from Alfa Aesar (Ward Hill, MA, USA). Foetal bovine serum (FBS), l-glutamine, Hank’s balanced salt solution (HBSS), trypsin-EDTA (ethylenediaminetetraacetic acid), nonessential amino acids solution (2 mM), penicillin/streptomycin solution (100 U/mL and 100 mg/mL, respectively), and DMEM (Dulbecco’s Modified Eagle Medium) were from Hyclone (Logan, UT, USA). Water was treated in a Milli-Q water purification system (TGI Pure Water Systems, Greenville, SC, USA).

### 3.3. Chemical Characterization of T. impetigosa Inner Bark

#### 3.3.1. Free Sugars

Free sugars were determined by high performance liquid chromatography coupled to a refraction index detector (HPLC-RI), after an extraction procedure previously described by the authors [28] using melezitoze as internal standard (IS). The equipment consisted of an integrated system with a pump (Knauer, Smartline system 1000, Berlin, Germany), degasser system (Smartline manager 5000), auto-sampler (AS-2057 Jasco, Easton, MD, USA), and an RI detector (Knauer Smartline 2300). Data were analyzed using Clarity 2.4 Software (DataApex, Podohradska, Czech Republic). The chromatographic separation was achieved with a Eurospher 100-5 NH2 column (4.6 × 250 mm, 5 µm, Knauer), operating at 30 °C (7971 R Grace oven). The mobile phase was acetonitrile/deionized water, 70:30 (*v*/*v*) at a flow rate of 1 mL/min. The compounds were identified by chromatographic comparisons with authentic standards. Quantification was performed using the internal standard method and sugar contents were further expressed in g per 100 g of dry weight (dw).

#### 3.3.2. Organic Acid

Organic acids were determined following a procedure previously described by the authors [29]. The analysis was performed using a Shimadzu 20A series UFLC (Shimadzu Corporation, Kyoto, Japan). Separation was achieved on a SphereClone (Phenomenex, Torrance, CA, USA) reverse phase C18 column (4.6 × 250 mm, 5 μm), thermostatted at 35 °C. The elution was performed with sulfuric acid 3.6 mM using a flow rate of 0.8 mL/min. Detection was carried out in a PDA, using 215 nm and 245 nm (for ascorbic acid) as preferred wavelengths. The organic acids found were quantified by comparison of the area of their peaks with calibration curves obtained from commercial standards of each compound. The results were expressed in g per 100 g of dry weight (dw).

#### 3.3.3. Fatty Acids

The fatty acids were determined by gas chromatography with flame ionization detection (GC-FID)/capillary column as described previously by the authors [28], and after methylation of the fatty acids (obtained after Soxhlet extraction using petroleum ether) with 5 mL of methanol:sulfuric acid:toluene 2:1:1 (*v:v:v*), during at least 12 h in a bath at 50 °C and 160 rpm; then 3 mL of deionized water were added, to obtain phase separation; the FAME were recovered with 3 mL of diethyl ether by shaking in a vortex, and the upper phase was passed through a micro-column of sodium sulfate anhydrous, in order to eliminate the water; the sample was recovered in a vial with Teflon, and before injection the sample was filtered with 0.2 µm nylon filter from Whatman. The analysis was carried out with a DANI model GC 1000 instrument (Contone, Switzerland) equipped with a split/splitless injector, a flame ionization detector (FID at 260 °C) and a Macherey–Nagel (Düren, Germany) column (50% cyanopropyl-methyl-50% phenylmethylpolysiloxane, 30 m × 0.32 mm i.d. × 0.25 μm df). The oven temperature program was as follows: the initial temperature of the column was 50 °C, held for 2 min, then a 30 °C/min ramp to 125 °C, 5 °C/min ramp to 160 °C, 20 °C/min ramp to 180 °C, 3 °C/min ramp to 200 °C, 20 °C/min ramp to 220 °C, and held for 15 min. The carrier gas (hydrogen) flow-rate was 4.0 mL/min (0.61 bar), measured at 50 °C. Split injection (1:40) was carried out at 250 °C. Fatty acid identification was made by comparing the relative retention times of FAME peaks from samples with standards. The results were recorded and processed using the CSW 1.7 Software (DataApex 1.7) and expressed in relative percentage of each fatty acid.

#### 3.3.4. Tocopherols

Tocopherols were determined following a procedure previously described by the authors [28]. Analysis was performed by HPLC (equipment described above), and a fluorescence detector (FP-2020; Jasco) programmed for excitation at 290 nm and emission at 330 nm. The chromatographic separation was achieved with a Polyamide II (4.6 × 250 mm, 5 µm) normal-phase column from YMC Waters operating at 30 °C. The mobile phase used was a mixture of *n*-hexane and ethyl acetate (70:30, *v*/*v*) at a flow rate of 1 mL/min. The compounds were identified by chromatographic comparisons with authentic standards. Quantification was based on the fluorescence signal response of each standard, using the IS (tocol) method and by using calibration curves obtained from commercial standards of each compound. The results were expressed in mg per 100 g of dry weight (dw).

### 3.4. Bioactivity Evaluation of T. impetigosa Extracts and Dietary Supplements

#### 3.4.1. Samples Preparation

The infusions were prepared from the dry inner bark (2 g) that was added to 200 mL of boiling distilled water and left to stand at room temperature for 5 min, and then filtered under reduced pressure. The methanolic extracts were obtained from the dry inner bark (4 g) that was extracted by stirring with 40 mL of methanol (25 °C at 150 rpm) for 1 h and subsequently filtered through Whatman No. 4 paper. The residue was then extracted with an additional 30 mL of methanol (25 °C at 150 rpm) for 1 h. The combined extracts were evaporated at 35 °C (rotary evaporator Büchi R-210, Flawil, Switzerland) to remove the methanol, and further redissolved in methanol (final concentration 20 mg/mL), for antioxidant activity evaluation, or water (final concentration 8 mg/mL) for cytotoxicity evaluation. The pills were powdered, diluted in 100 mL of water (final concentration 5 mg/mL) and vacuum filtered. The syrup was diluted in water in order to obtain a final extract concentration of 0.94 mg/mL (working concentration for the bioactive assays).

The final solutions obtained were further diluted to different concentrations to be submitted to distinct in vitro bioassays.

#### 3.4.2. Antioxidant Properties

The antioxidant activity of the samples was evaluated by DPPH radical-scavenging activity, reducing power, inhibition of β-carotene bleaching and inhibition of lipid peroxidation using TBARS in brain homogenates [30]. DPPH radical-scavenging activity was evaluated by using an ELX800 microplate Reader (Bio-Tek Instruments, Inc.; Winooski, VT, USA), and calculated as a percentage of DPPH discolouration using the formula: [(ADPPH-AS)/ADPPH] × 100, where AS is the absorbance of the solution containing the sample at 515 nm, and ADPPH is the absorbance of the DPPH solution. Reducing power was evaluated by the capacity to convert Fe^3+^ into Fe^2+^, measuring the absorbance at 690 nm in the microplate Reader mentioned above. Inhibition of β-carotene bleaching was evaluated though the β-carotene/linoleate assay; the neutralization of linoleate free radicals avoids β-carotene bleaching, which is measured by the formula: β-carotene absorbance after 2 h of assay/initial absorbance) × 100. Lipid peroxidation inhibition in porcine brain homogenates was evaluated by the decreasing in thiobarbituric acid reactive substances (TBARS); the color intensity of the malondialdehyde-thiobarbituric acid (MDA-TBA) was measured by its absorbance at 532 nm; the inhibition ratio (%) was calculated using the following formula: [(A − B)/A] × 100%, where A and B were the absorbance of the control and the sample solution, respectively. The results were expressed in EC_50_ values (sample concentration providing 50% of antioxidant activity or 0.5 of absorbance in the reducing power assay; mg extract/mL solution). Trolox was used as positive control [30].

#### 3.4.3. Total Antioxidants

Total phenolics were estimated by Folin-Ciocalteu colorimetric assay according to procedures previously described [31] and the results were expressed as mg of gallic acid equivalents (GAE) per g of extract in each sample. Total flavonoids were determined by a colorimetric assay using aluminum trichloride, following procedures previously reported [29]; the results were expressed as mg of (+)-catechin equivalents (CE) per g of extract in each sample.

#### 3.4.4. Cytotoxic Properties

Four human tumor cell lines were used: MCF-7 (breast adenocarcinoma), NCI-H460 (non-small cell lung cancer), HeLa (cervical carcinoma) and HepG2 (hepatocellular carcinoma). Cells were routinely maintained as adherent cell cultures in RPMI-1640 medium containing 10% heat-inactivated FBS and 2 mM glutamine (MCF-7 and NCI-H460) or in DMEM supplemented with 10% FBS, 2 mM glutamine, 100 U/mL penicillin, and 100 mg/mL streptomycin (HeLa and HepG2 cells), at 37 °C, in a humidified air incubator containing 5% CO_2_. Each cell line was plated at an appropriate density (7.5 × 10^3^ cells/well for MCF-7 and NCI-H460 or 1.0 × 10^4^ cells/well for HeLa and HepG2) in 96-well plates. Sulforhodamine B assay was performed according to a procedure previously described by the authors [32].

For hepatotoxicity evaluation, a cell culture was prepared from a freshly harvested porcine liver obtained from a local slaughter house, according to a procedure established by the authors [32]; it was designed as PLP2. Cultivation of the cells was continued with direct monitoring every two to three days using a phase contrast microscope. Before confluence was reached, cells were subcultured and plated in 96-well plates at a density of 1.0 × 10^4^ cells/well, and commercial in DMEM medium with 10% FBS, 100 U/mL penicillin and 100 μg/mL streptomycin.

The results were expressed in GI_50_ values (sample concentration that inhibited 50% of the net cell growth; µg extract/mL solution). Ellipticine was used as positive control.

### 3.5. Statistical Analysis

For all the experiments four samples were analyzed and all the assays were carried out in triplicate. The results are expressed as mean values and standard deviation (SD). The differences between the different samples were analyzed using one-way analysis of variance (ANOVA) followed by Tukey’s honestly significant difference post hoc test with α = 0.05. This treatment was carried out using SPSS v. 22.0 program (IBM Corporation, Armonk, NY, USA).

## 4. Conclusions

Overall, this work allowed the evaluation of the bioactivity of *T. Impetigosa* based phytopreparations (methanolic extract and infusion) and phytoformulations (two dietary supplements namely, pills and syrup). The syrup and the methanolic extract revealed the highest antioxidant activity, while the infusion revealed the lowest activity, results correlated with the corresponding phenolic content. The methanolic extract was the only sample showing inhibitory effects against all the tested human tumor cell lines, with a stronger effect on NCI-H460. This highlights the importance of using methanol in the extraction of the bioactive compounds. However, none of the samples revealed toxicity in non-tumor cells (PLP2). *T. Impetigosa* was revealed to be a rich source of nutrients and bioactive compounds, with potential to be used in alternative medicine using specific formulations for antioxidant or cytotoxic purposes.
